# The Membrane Interaction of Alpha-Synuclein

**DOI:** 10.3389/fncel.2021.633727

**Published:** 2021-03-04

**Authors:** Cencen Liu, Yunfei Zhao, Huan Xi, Jie Jiang, Yang Yu, Wei Dong

**Affiliations:** ^1^Key Laboratory of Medical Electrophysiology of Ministry of Education and Medical Electrophysiological Key Laboratory of Sichuan Province, Institute of Cardiovascular Research, Southwest Medical University, Luzhou, China; ^2^Department of Histology and Embryology, School of Basic Medical Sciences, Southwest Medical University, Luzhou, China; ^3^Department of Neurosurgery, Affiliated Hospital of Southwest Medical University, Luzhou, China; ^4^Neurosurgical Clinical Research Center of Sichuan Province, Luzhou, China

**Keywords:** Parkinson's disease, alpha-synuclein, membrane, SNAREs, synaptic transmission

## Abstract

A presynaptic protein closely related to Parkinson's disease (PD), α-synuclein (α-Syn), has been studied extensively regarding its pathogenic mechanisms. As a physiological protein in presynapses, however, α-Syn's physiological function remains unclear. Its location in nerve terminals and effects on membrane fusion also imply its functional role in synaptic transmission, including its possible interaction with high-curvature membranes *via* its N-terminus and amorphous C-terminus. PD-related mutants that disrupt the membrane interaction (e.g., A30P and G51D) additionally suggest a relationship between α-Syn's pathogenic mechanisms and physiological roles through the membrane binding. Here, we summarize recent research on how α-Syn and its variants interact with membranes and influence synaptic transmission. We list several membrane-related connections between the protein's physiological function and the pathological mechanisms that stand to expand current understandings of α-Syn.

## Introduction

**Figure 1:**
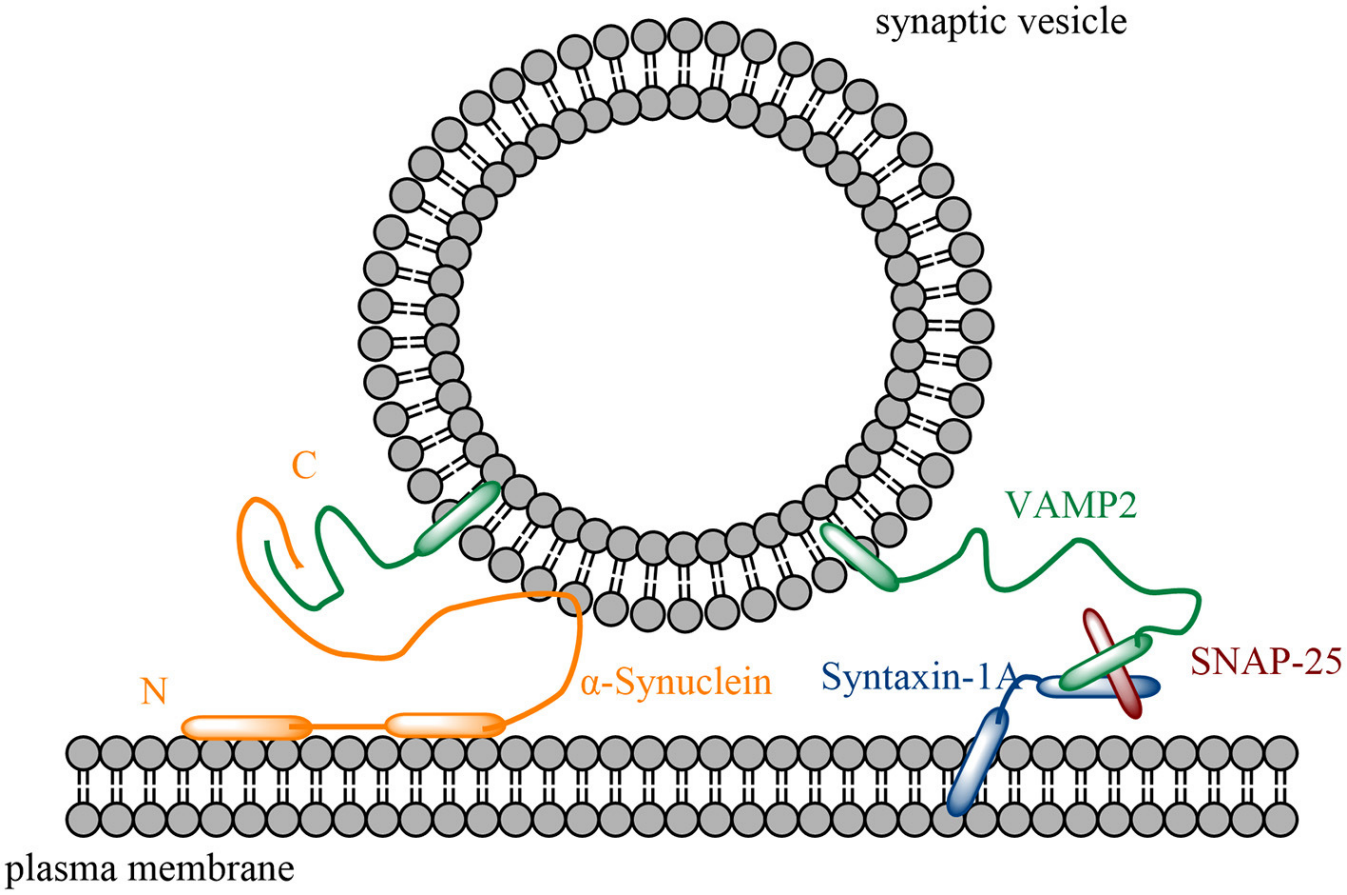
Schematic diagram of α-Syn in SNARE (a complex composed of syntaxin-1A, VAMP2 and SNAP-25)-mediated membrane interaction. α-Syn's N-terminus forms two helices to interact with the plasma membrane, while α-Syn interacts with VAMP2 at the C-terminus.

The protein α-Synuclein is highly soluble and plays a central role in the pathogenesis in Parkinson's disease and other synucleinopathies. With only 140 amino acids, α-Synuclein (α-Syn) is a small peripheral membrane protein that localizes specifically to the axon terminal in neurons (Maroteaux et al., 1988; George et al., 1995; Iwai et al., 1995; Bendor et al., 2013). In α-Syn's unusual, unique structure (Figure 1), its highly conserved N-terminus contains seven 11-mer repeats (residues 1–95) with the KTKEGV consensus sequence, similar to apolipoprotein, that forms three turns of an amphipathic α-helix and mediates α-protrusion associated with membranes of synuclein and lipids (Davidson et al., 1998; Eliezer et al., 2001; Bussell and Eliezer, 2003; Chandra et al., 2003; Bussell et al., 2005). Strangely, all identified mutations related to synucleinopathies (e.g., A30P, E46K, H50Q, G51D, A53E, and A53T) are located in the N-terminal domain (Polymeropoulos et al., 1997; Krüger et al., 1998; Zarranz et al., 2004; Appel-Cresswell et al., 2013; Lesage et al., 2013; Proukakis et al., 2013; Pasanen et al., 2014). Beyond that, five of them are clustered in eight residues, which suggests the potential for a pathology of lipid binding or even a lack of lipid binding to α-Syn. Aside from that domain, the NAC domain (i.e., residues 60–95) is responsible for α-Syn's aggregation (Uéda et al., 1993) and aids the detection of lipid properties (Fusco et al., 2014). Meanwhile, the C-terminus (i.e., residues 96–140), a highly acidic and largely unstructured domain (Davidson et al., 1998; Bussell and Eliezer, 2003; Ulmer et al., 2005), is the target of various post-translational modifications (Oueslati et al., 2010). It is also considered to bind to proteins, ions, polycations, and polyamines (Paik et al., 1999; Nielsen et al., 2001; Fernández et al., 2004; Brown, 2007), as well as to modulate α-Syn's binding to membranes (Sevcsik et al., 2011) and protect it from aggregation (Crowther et al., 1998; Park et al., 2002; Hoyer et al., 2004).

Located in presynapses, α-Syn could interact with synaptic vesicles and act as a molecular chaperone of soluble N-ethylmaleimide-sensitive factor attachment protein receptor (SNARE) complexes (George et al., 1995). Although such evidence indicates α-Syn's role in neurotransmitter release and synaptic plasticity, its precise function remains unclear. α-Syn is also absent in worms, fruit flies, and yeasts, which suggests that it is not generally required for synaptic transmission or membrane trafficking (Abeliovich et al., 2000; Chandra et al., 2004; Burre et al., 2010; Jensen et al., 2011). Therefore, given existing research, we have summarized the current knowledge about the interaction of α-Syn with membranes, how variants of α-Syn affect the interaction with membranes, and how α-Syn affects synaptic transmission. We also discuss the possible mechanism of the transition between physiological function and pathological mechanism.

## Physiological Mechanism of Membrane Fusion

As a basic life process, membrane fusion involves subcellular compartmentation, cell growth, hormone secretion, and neurotransmission (Wickner and Schekman, 2008). Membrane fusion begins with the gradual fusion of the outer and inner leaflets between two membranes, which causes lipids and other contents (e.g., proteins, glycoprotein, and glycolipid) to mix. From there, it consists of multiple distinct stages, including tethering, docking and priming, hemifusion, and full fusion (Tian et al., 2019).

Critical evidence concerning proteins active in membrane fusion has accumulated in the literature to date. Notably, SNAREs are considered to represent core fusion elements that assembled in a four-helix bundle structure, releasing energy such that the membranes enter into close proximity and eventually undergo membrane fusion (Sutton et al., 1998). Proteins such as α-Syn and cysteine string protein α (CSPα) affect SNARE complexes, while the interaction between lipid molecules and proteins plays a key role in regulating membrane fusion (Brunger et al., 2015; Wang et al., 2016b; Bao et al., 2018; Das et al., 2020).

### α-Syn's Interaction With Membranes

In view of its enriched expression in neuronal cells, many endogenous roles for α-Syn have been proposed, including ones that involve controlling synaptic vesicle release (Snead and Eliezer, 2014), modulating secretory pathways (Wang and Hay, 2015), and even regulating vesicle transport (Jensen et al., 1998). Another well-described biochemical property of α-Syn is membrane binding associated with structural switching.

### α-Syn's Function in Synaptic Transmission

*In vitro* research has shown that α-Syn inhibits membrane fusion by stabilizing the lipid packing of stressed bilayers independently of other protein factors that may be involved in the fusion machinery of membranes (Kamp et al., 2010). At the same time, α-Syn was found to significantly promote the clustering of protein-reconstituted liposomes that mimic synaptic vesicles, albeit with little effect on Ca^2+^-triggered fusion in a single vesicle-vesicle system with reconstituted neuronal SNAREs, synaptotagmin-1, and complexin-1 (Diao et al., 2013). It was also found to participate in vesicle aggregation, initiation, fusion (i.e., assembly of SNARE complex), and recycling (i.e., disassembly of SNARE complex), all of which relate to membrane fusion (Lashuel et al., 2013).

Studies have shown that α-Syn functions within various aspects of synaptic transmission. For one, it promotes the assembly of SNARE complexes by directly interacting with N-terminus of VAMP2 through the highly flexible, negatively charged C-terminal region (Burre et al., 2010). For another, via its N-terminal region, α-Syn's membrane anchoring is also essential to the process (Wang et al., 2016a). α-Syn may also cooperate with CSPα to maintain SNARE proteins assembly and neurotransmission (Hou et al., 2017). Additional evidence suggests that native α-Syn does not compromise the efficiency of synaptic vesicle exocytosis but does help to increase the availability of synthetic vesicles at the synapse (Diao et al., 2013). Recently, studies show both VAMP2 and synapsin cooperate to promote clustering of SVs and regulating SV recycling with different mechanisms (Atias et al., 2019; Sun et al., 2019). However, other research has indicated that α-Syn knockout exerts little effect on synaptic transmission (Nemani et al., 2010) and that α-Syn's overexpression reduces the release of neurotransmitters by disrupting vesicle docking in exocytosis (Larsen et al., 2006). Further still, because α*βγ*-synuclein triple-knockout mice lacking synucleins developed age-dependent neurological impairments, exhibited decreased SNARE-complex assembly, and died prematurely, synucleins may also sustain normal SNARE complex assembly in presynaptic terminals during aging (Burre et al., 2010).

Other research has revealed that the promotive effect of α-Syn on SNARE-dependent bilayer merging mainly occurs via the enhancement of vesicle docking (Hawk et al., 2019). Recent work also revealed no significant changes observed in merging efficiency, the ratio of instant-to-delayed merger events, or the kinetics of bilayer merging other than the frequency of vesicle docking, especially since α-Syn stimulates vesicle docking without altering the dynamics of bilayer mergers in lipid mixing (Hawk et al., 2019). To that, researchers have contributed the hypothesis that α-Syn binds to vesicle-associated membrane protein 2 (VAMP2) by using its unstructured C-terminus, while simultaneously interacting with the target plasma membrane via its amphipathic N-terminal region, thereby aiding the recruitment of synaptic vesicles to the plasma membrane. The α-Syn's C-terminus is critical to promoting vesicle docking, because after truncating the C-terminus, the docking process of vesicles was inhibited, and overexpression had destroyed the accumulation of vesicles within the synapse (Lou et al., 2017). On the contrary, it had been found that the inhibition of docking by α-Syn is coupled with the α-Syn's membrane binding but not with the interaction with VAMP2, although the fusion inhibition by α-Syn oligomers at much lower concentrations requires α-Syn's interaction with VAMP2 (Lai et al., 2014). Once aggregated, the multivalent oligomeric species containing multiple binding sites can bind to VAMP2 on the vesicle, which renders VAMP2 unable to interact with t-SNARE (i.e., syntaxin-1A and SNAP-25) on the plasma membrane (Choi et al., 2013) and thus severely reduces the possibility of bilayer merging. In addition, some convincing evidence suggests that α-Syn promotes the filling of vesicles by directly interacting with and modulating vesicular monoamine transporter 2 and the reuptake of dopamine *via* the dopamine transporter (Lee et al., 2001; Wersinger and Sidhu, 2003; Guo et al., 2008; Swant et al., 2011; Butler et al., 2015). Latest studies also suggest that α-Syn can promote endocytosis by increasing phosphatidylinositol 4,5-bisphosphate level (Schechter et al., 2020). Those findings may stimulate new ideas for further examination of the process of synaptic transmission.

### Fusion Pore Regulation

In general, fusion begins with the formation of a narrow pore called a *fusion pore* that allows water, solute, and membrane to move between compartments (Brose et al., 2019). A fusion pore may grow, contract, or close, all under the influence of both mechanical forces and biological cues (Brose et al., 2019). In that context, α-Syn's aggregation has been shown to cause the formation of oligomeric intermediates that interact with membranes to form fusion pores, while α-Syn itself seems to partly accelerate the opening of the pores (Logan et al., 2017). During exocytosis, synaptic vesicles also form fusion pores that dilate before fully collapsing into the plasma membrane. However, because fusion pores can also reclose during kiss-and-run events in which vesicles are immediately regenerated (Alabi and Tsien, 2013), the regulation of the membrane curvature may affect the behavior of fusion pores. If so, then because α-Syn can bind to anionic membranes with high curvatures (Davidson et al., 1998; Jensen et al., 2011; Pranke et al., 2011), overexpression of α-Syn has been shown to influence the behavior of exocytotic fusion pores. The dilation of fusion pores would thus be expected to limit the release of neuromodulators that dissociate slowly from luminal matrices (e.g., monoamines and peptides), but without affecting classical transmitters such as glutamate that can rapidly escape, even through small pores. However, α-Syn has increased the number of SNARE complexes (Burre et al., 2010), which may also heighten the force that drives the dilation of fusion pores and thus promotes cargo release (Shi et al., 2012). In view of those dynamics, it is difficult to reconcile the observed inhibition of cargo release and the notion that α-Syn chaperones SNARE complexes. Altogether, α-Syn appears to play a dual role. On the one hand, it inhibits membrane fusion *in vivo* and *in vitro* by directly acting on lipid bilayers. On the other hand, it may promote the accumulation of SNARE complexes by inhibiting exocytosis, thereby preventing the disassembly of complexes present on vesicles primed for fusion (Kamp et al., 2010, Nuscher et al., 2004; Braun and Sachs, 2015).

### Modulation With Ca^2+^

The primary location of α-Syn is at the presynaptic terminal, where calcium fluctuations can occur in concentrations in the hundreds of μM. It is well established that calcium ions play an important role in triggering synaptic transmission, and studies have shown that calcium binds to α-Syn at its C-terminus, where Ca^2+^ can regulate the role of α-Syn and the plasma membrane. *In vitro* experiments have confirmed that different concentrations of calcium ions exert different effects on proteins in plasma membranes (e.g., presynaptic membrane), and it is well known that cytosolic Ca^2+^ regulates vesicle docking, priming, fusion, and the expansion of fusion pores (Man et al., 2015). Still other work has shown that α-Syn interacts with plasma membranes in a specific structure and affects calcium signal transduction, while β-sheet-rich-poly α-Syn can cause Ca^2+^ deregulation and Ca^2+^-dependent cell death (Angelova et al., 2016). Added to that, a recent study has demonstrated α-Syn's novel effects on mobilizing the release of Ca^2+^ from thapsigargin-sensitive Ca^2+^ pools to enhance the ATP-induced increase of Ca^2+^ concentration, which enhances vesicle fusion. At the same time, soluble α-Syn elevates the same release of Ca^2+^ from thapsigargin-sensitive Ca^2+^ pools to enhance ATP-induced fusion, which reveals α-Syn's novel role in coupling vesicles to specific Ca^2+^ microdomains. By contrast, aggregated α-Syn, in a Ca^2+^-independent pathway, inhibits vesicle priming but does not affect the dilation of fusion pores (Huang et al., 2018).

## Influence of α-Syn's Different Truncated Ends on Membrane Interaction

α-Syn has an N-terminal membrane-binding region that binds to phospholipid bilayers and a C-terminal region that interacts with VAMP2 (Lou et al., 2017). *Via* its N-terminus and amorphous C-terminus, α-Syn can also interact with high-curvature membranes. At both ends, however, truncation affects α-Syn's interaction with membranes to varying degrees, hence the interest among researchers in analyzing how α-Syn's different truncated ends affect membrane interaction.

Several studies have furnished support for an emerging view that α-Syn's N-terminal region plays an anchoring role in membrane interaction, namely by modulating α-Syn's physiological as well as pathological role (Diao et al., 2013; Fusco et al., 2016; O'Leary and Lee, 2019). Since then, additional research has indicated that approximately 14 N-terminal residues enter anionic membranes at a skewed angle of insertion, which relates to the helical region's folding onto the membrane's surface, thereby synergistically establishing the joint α-Syn–lipid structures (Cholak et al., 2020). Meanwhile, the deletion of residues 2–14 reduces α-Syn's membrane localization in mammalian cells, which indicates that the N-terminal anchor exerts an impact *in vivo*. All of that evidence shows that avidity within the N-terminal anchor couples N-terminal insertion and helical surface binding, both of which are crucial for α-Syn's interaction with membranes and cellular localization and may even affect membrane fusion (Cholak et al., 2020). Fusion has also proven to fail with a truncated α-Syn due to its lacking the charged C-terminal domain (Kamp et al., 2010). Last, genomic editing to disrupt α-Syn's N-terminal domain, which is important for membrane association, induced mitochondrial elongation without changes in fusion-fission protein levels, thereby suggesting that αSyn plays a direct physiological role in maintaining the size of mitochondria (Pozo Devoto et al., 2017).

## Influence of Mutants on Membrane Interaction

Studies have additionally revealed that α-Syn factors into neurodegenerative disorders (Logan et al., 2017) and that various secondary structures of α-Syn are involved in physiological and pathological processes (El-Agnaf et al., 1998; Conway et al., 2000; Lashuel et al., 2002; Uversky, 2007; Burré et al., 2014, 2015; Wang et al., 2014). For those reasons, knowing the effect of Parkinson's disease-related mutants that disrupt membrane interaction is important to understanding the possible relationship between pathogenic mechanisms and physiological roles at play in membrane binding. Research has shown that the A53T mutation had the highest membrane affinity with wild-type α-Syn, even compared to A30P (Perlmutter et al., 2009). Meanwhile, the increased affinity of the E46K mutant for vesicles containing negatively charged lipids has also been observed to induce an additional hydrogen bond between the protein and either the detergent or the lipid. Even so, the literature on those topics remains slim, and other possible mechanisms of the effects of PD-related mutants indeed warrant further investigation.

## Discussion

As a type of membrane-based interaction, membrane fusion is vital to the release of neurotransmitters and plays a chief role in transmitter mechanisms. As a protein that can regulate membrane fusion, α-Syn is essential in the normal function of synapses, especially in synaptic transmission. Since changes in α-Syn can affect its function of regulating membrane fusion and thus affect synaptic transmission—and its changes may also contribute to some neurodegenerative diseases—a more profound understanding of α-Syn is critically needed.

Physiologically, as a chaperone, α-Syn participates in the assembly of SNARE complexes and may perform other functions as well. Pathologically, by contrast, α-Syn misfolds into neurotoxic aggregates that mediate neurodegeneration and propagate between neurons (Burré et al., 2014). Synuclein also forms a perplexing web of interactions with lipids, trafficking machinery, and other regulatory factors (Wang and Hay, 2015). Moreover, the protein's effects on endocytosis, exocytosis, and vesicle circulation are closely related to synaptic transmission. In the central nervous system, the release of calcium-dependent neurotransmitters regulates the roles of α-Syn and membrane, which affect not only the interaction between α-Syn and synaptic vesicles, but also neurotransmission, a qualitative release process. Therefore, the aggregation of α-Syn may relate to α-Syn's toxic effect, which stands to inspire new ideas for drug targets able to prevent that effect. On top of that, other experiments could study the effect of PD-related mutants on fusion to provide insights into the treatment of neurodegenerative diseases more generally.

## Author Contributions

WD, CL, and YZ conceived the perspective of the work. CL and YZ drafted the manuscript. CL designed the figure. All authors revised and approved the final version of the manuscript.

## Conflict of Interest

The authors declare that the research was conducted in the absence of any commercial or financial relationships that could be construed as a potential conflict of interest.
